# Linking neuroscientific research on decision making to the educational context of novice students assigned to a multiple-choice scientific task involving common misconceptions about electrical circuits

**DOI:** 10.3389/fnhum.2014.00014

**Published:** 2014-01-27

**Authors:** Patrice Potvin, Élaine Turmel, Steve Masson

**Affiliations:** Université du Québec à MontréalMontréal, QC, Canada

**Keywords:** uncertainty, certainty, misconception, electricity, fMRI, decision making, education

## Abstract

Functional magnetic resonance imaging was used to identify the brain-based mechanisms of uncertainty and certainty associated with answers to multiple-choice questions involving common misconceptions about electric circuits. Twenty-two scientifically novice participants (humanities and arts college students) were asked, in an fMRI study, whether or not they thought the light bulbs in images presenting electric circuits were lighted up correctly, and if they were certain or uncertain of their answers. When participants reported that they were unsure of their responses, analyses revealed significant activations in brain areas typically involved in uncertainty (anterior cingulate cortex, anterior insula cortex, and superior/dorsomedial frontal cortex) and in the left middle/superior temporal lobe. Certainty was associated with large bilateral activations in the occipital and parietal regions usually involved in visuospatial processing. Correct-and-certain answers were associated with activations that suggest a stronger mobilization of visual attention resources when compared to incorrect-and-certain answers. These findings provide insights into brain-based mechanisms of uncertainty that are activated when common misconceptions, identified as such by science education research literature, interfere in decision making in a school-like task. We also discuss the implications of these results from an educational perspective.

## INTRODUCTION

Answering scientific questions correctly has always been a considerable challenge for students and an important – and sometimes exclusive – indicator of academic success. One of the reasons why it is such a challenge is that students sometimes have “misconceptions” about natural phenomena that interfere with learning and, therefore, divert students from giving correct answers. Misconceptions, as typically defined in the science education research tradition, are understood as “deeply rooted” ideas or common erroneous beliefs about how nature works that are “not in harmony with the science views or are even in stark contrast to them” (Duit and Treagust, 2003). For example, people often believe that heavier balls fall more rapidly, even in the absence of air resistance (Brown and Hammer, 2008), or they believe that a bulb can be lighted up with a battery and a single wire (Sencar and Eryilmaz, 2004; Pesman and Eryilmaz, 2010). These misconceptions are problematic for science education because they are known to be very difficult to change. Contrary to the widespread belief that students produce wrong answers because of their “lack of knowledge,” the misconception paradigm is interested in the existence of a form of personal or socially shared knowledge that does not conform to scientifically established knowledge and that can explain the recurrence or persistence of some wrong answers (Potvin, 2013).

School science, as well as educational researchers, often uses multiple-choice (or true/false) questions to assess students’ performances or to diagnose misconceptions. Indeed, multiple-choice questions often include wrong answers that intentionally contain misconceptions in order to test students’ knowledge of the material. In such contexts, the production of answers becomes a matter of decision making. Indeed, decision making can be defined as the “process of making choices or reaching conclusions” (Paulus, 2005). However, such a process can be very complex and sometimes decisions must be made under varying degrees of uncertainty.

Indeed, in previous research, we have been able to show that subjects’ expression of certainty/uncertainty about their own conceptions can have a very important effect on subsequent learning in science (Potvin et al., 2010), perhaps even more than previous knowledge. Since wrong answers might not always the product of a lack of knowledge, it is important to study all the possible combinations of certainty and accuracy, including the ones where participants have conceptions and misconceptions on which they can base their feelings of certainty. Indeed, many science education papers have made it clear that the level of certainty/uncertainty associated with the production of an answer is crucial to the qualification of conceptions and misconceptions (Hasan et al., 1990; Merenluoto and Lehtinen, 2002; Potgieter et al., 2010; Caleon and Subramanian, 2012). Thus, when wrong answers are nevertheless associated with a high degree of certainty, they usually indicate the presence of misconceptions.

Inspired by these research efforts, particularly Hasan et al.’s (1990), we have proposed four categories of answers, based on accuracy and expressed certainty/uncertainty (Potvin et al., 2010). The first category includes all correct-and-certain answers. We call this category *Legitimate Certainty* (LC). It can be suggested that in a task where the distractors are misconceptions, these answers might indicate that subjects do not believe in the considered misconceptions, or that they have been able to make more scientific conceptions prevail. The second category includes all incorrect yet nevertheless certain answers. We call this category *Over-Estimation* (OE). According to Hasan et al. (1990), these answers might indicate the presence of misconceptions more than answers for which doubt is expressed. Indeed, whether or not they are correct [Underestimation (UE)] or incorrect [Legitimate Doubt (LD)], answers that are associated with a feeling of doubt can, according to Hasan, be attributed to a lack of knowledge rather than to the presence of a conception on which subjects base their answer, whether or not it is scientifically accurate.

This particular categorization will allow us to formulate two research questions that have the potential to help us better understand, from a neuroscientific point of view, the ordinary educational context of novice students assigned to a multiple-choice task involving misconceptions in electricity. The first question will address the issue of differences that exist between certain and uncertain answers. The results will allow us to establish interesting links between studies that have been conducted with other kinds of tasks. The second question will address the issue of the difference between cases where misconceptions most likely prevailed (OE) and cases where they were overcome (LC).

### DECISION MAKING UNDER CERTAINTY/UNCERTAINTY

Considered as a decision-making process, the educational event of producing an answer to a multiple-choice question can be linked to other research in the field and can therefore be used as a starting point. However, according to Volz et al. (2005), understanding the mechanisms by which decisions are made is of very recent interest in neurobiology, and “we are far away from providing a single general theory of human decision making […]. But incorporating and bringing together the findings from different disciplines concerning different aspects of decision-making will help in understanding the big picture” (p. 403). Indeed, even if this “big picture” is not clear yet, a considerable number of research efforts have studied the general brain-based mechanisms associated with decision making under uncertainty using a variety of tasks.

In most studies, the experimenters control the level of uncertainty by setting the level of ambiguity (Huettel et al., 2005; Grinband et al., 2006; Simmons et al., 2008; Daniel et al., 2010). In other studies, control of the level of uncertainty is modulated by the probability of events to occur (Critchley et al., 2001; Volz et al., 2003; Krain et al., 2006; Tobler et al., 2007; Preuschoff et al., 2008; Schlosser et al., 2009; Hosseini et al., 2010; Sarinopoulos et al., 2010; Stern et al., 2010).

However, since typical school science questions about concepts are not ambiguous, and since their formulation usually excludes uncertain probabilities of events to occur, we will concentrate on studies interested in uncertainty levels that are determined by internal lacks of knowledge. Volz et al. (2004, 2005), for example, used two experiments to explore “variants of uncertainty.” In one experiment, they used a task where subjects were informed to varying degrees about the rules controlling the environment, which created different levels of lack of knowledge and, therefore, uncertainty. A parametric analysis revealed the activation of the posterior fronto-median cortex (BA8) “as a common cortical substrate of uncertain decisions” (p. 409). These results are interesting to us because they address the issue of uncertainty induced by a lack of knowledge, but they do not address the question of the accuracy of predictions, which is rather important in educational contexts.

In a functional magnetic resonance imaging (fMRI) study (Harris et al., 2008), subjects were asked if some statements (about mathematics, geography, biography, and religion, among other subjects) were true, false, or undecidable. The state of uncertainty, when compared with “independent of context” states of belief and disbelief (p. 143), was found to be associated with the anterior cingulate and decrease the caudate signal. This result is interesting to us because many of the questions were very similar to the school question format, like “(2 + 6) + 8 = 16,” “Eagles are common pets,” or “Senegal borders Guinea” (p. 142). The state of uncertainty was associated with activation of the anterior cingulate as well as the superior frontal gyrus.

Other studies have preferred to use memory recognition tasks where subjects reported different levels of confidence in their recollections of previously learned information, such as words or faces (Henson et al., 2000; Chua et al., 2006, 2009; Kim and Cabeza, 2009; Hayes et al., 2011). In some instances, high confidence was associated with the anterior and posterior cingulate, medial frontal, and medial temporal lobe (MTL) activations. However, it is highly likely that these results might have been due to the particular nature of the task (face recognition), and therefore it is not unusual that areas such as the MTL, usually associated with memory functions, were more activated.

These studies bear, to our knowledge, the closest resemblance to school tasks that aim at assessing or developing conceptual knowledge and can better enlighten the process of students resolving a conceptual task. They will therefore serve as a basis for formulating our certainty/uncertainty hypotheses. However, since they have not tested certainty issues when misconceptions are involved, a comparison with their results will be of interest. Therefore, hypothesis No.1 (Uncertainty > Certainty) includes the activation of posterior fronto-median cortex (BA8; Volz et al., 2004, 2005) and the anterior cingulate as well as the superior frontal gyrus (Harris et al., 2008). For Certainty > Uncertainty, it is more difficult to formulate hypotheses since many studies support a *modality specific* view on conceptual representations (Kiefer and Pulvermüller, 2012). Therefore results might be closely linked to the particular nature of the task used. Since the one we have used has not been tested before, proposing clear hypotheses at this stage might be a little reckless. Nevertheless, we will propose speculative discussion elements at the end of the article and comparisons with other research.

### DIFFERENCE BETWEEN OVER-ESTIMATION AND LEGITIMATE CERTAINTY

Recent research efforts have argued that for many kinds of scientific learning, the executive function of inhibition might play a very important role, as in conservation problems (Houdé, 2000; Houdé et al., 2011), or even in “misconceptual” problems (Babai et al., 2012; Shtulman and Valcarel, 2012). In fMRI studies conducted by Dunbar et al. (2007), Masson et al. (in press), Potvin (2013), and Nelson et al. (2007) with tasks involving electricity, mechanics, and chemistry misconceptions, it has been shown that experts activate regions that are generally associated with the function of inhibition. This suggests that misconceptions are not eradicated, abandoned, or restructured during the development of expertise (as is usually suggested in the epistemology-based conceptual change tradition), but rather are suppressed, allowing a prevalence of more scientific ideas (Potvin, 2013). The identified regions are the ventrolateral and dorsolateral prefrontal cortices, and sometimes the anterior cingulate. In our analysis, we will hypothesize (hypothesis No.2) that contrasts between LC and OE (LC > OE) will show activations of this kind. Thus it will be interesting to see if correct-and-certain answers (LC) given by novices show activations that are similar to correct answers given by experts. We will also check, using a *post hoc* analysis, for interesting activations in other possible contrasts.

### CHOICE OF MISCONCEPTIONS TO INCLUDE IN THE TASK

In order to test our hypotheses, we have chosen to study the pedagogical context of learning about electricity because misconceptions about basic electric phenomena have been studied thoroughly in the past and are therefore well known by the science education community. We have thus developed a cognitive task involving electric circuits that was based on educational research on students’ misconceptions regarding this topic (e.g., Shipstone, 1988; Sencar and Eryilmaz, 2004; Periago and Bohigas, 2005; Çepni and Keleş, 2006; Pesman and Eryilmaz, 2010; Potvin et al., 2010). The findings of this study will therefore provide insights into the brain-based mechanisms of uncertainty related to common misconceptions in science (electricity). It is our hope that this research will contribute to establishing a link between neuroscientific considerations and – at least – one authentic educational context, rather than establishing that link by using tasks of greater psychometrical value, but of less “ecological” (real life) value. If we succeed in establishing a few modest but convincing connections between decision making research and educational contexts, such as the assignment of scientific tasks involving misconceptions, then it could also be possible to begin considering some of the practical recommendations and prescriptions that emerge from the abounding decision making field as potentially interesting for educational purposes (teaching and learning).

## MATERIALS AND METHODS

### PARTICIPANTS

Twenty-three right-handed participants took part in the study. The images from 1 participant were excluded from the analysis due to a technical problem with the response box. Therefore, the images from 22 participants (11 males, between the ages of 18 and 20; *M* = 18.5; SD = 0.7) were used in the data analysis. Two functional series (out of four) from 1 of these 22 participants were also excluded from the analysis due to a trigger problem.

Since we needed “novice” participants who were less likely to have prior knowledge of electricity, we recruited humanities and arts college students who had never taken optional science courses during their studies. Since individuals with anxiety disorders may have different brain activations under uncertainty in the frontal and limbic regions (Krain et al., 2008), volunteers who met the criteria of the *Diagnostic and Statistical Manual of Mental Disorders: Fourth Edition, Text Revised* (DSM-IV-TR) regarding anxiety disorders were identified by the ADIS IV-R test (Newman et al., 2003) and excluded from the study (three women and six men out of a total of 32 volunteers were excluded for this reason). The remaining selected participants reported no abnormal neurological history (depression, schizophrenia, anxiety disorder, etc.). They received $20 for their participation and $20 as a transportation fee. Written informed consent for all participants was obtained prior to the experiment, and the study was approved by a local ethics committee (*Comité D’Éthique Mixte de la Recherche de l’Institut Universitaire de Gériatrie de Montréal, Canada*).

### TASK

**FIGURE 1:**
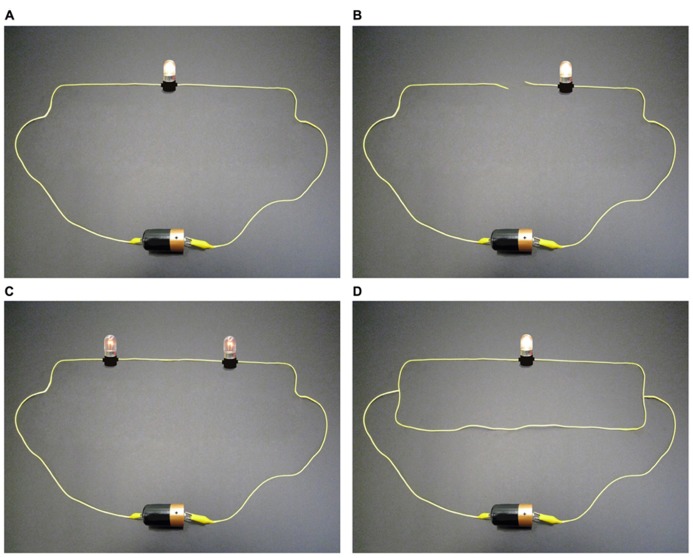
**A few examples of the electric circuits depicted in the experiment.** Scientifically correct and incorrect circuits were presented and, for each one, participants had to indicate, based on their own opinion, whether or not the circuit was correct and express their level of certainty/uncertainty about their answer. **(A)** Correct; **(B)** Incorrect; **(C)** Correct; **(D)** Incorrect.

Based on a number of educational studies emphasizing the typical difficulties surrounding the understanding of simple electric circuits (e.g., Shipstone, 1988; Periago and Bohigas, 2005; Çepni and Keleş, 2006;, 2006; Potvin et al., 2010), we produced a bank of images (Turmel et al., 2012) of series, parallel, and mixed electric circuits, each composed of wires, a battery, and bulbs (one, two, or three bulbs that could be either off or on at a low or a high level of brightness). The images consisted of photographs of real bulbs, wires, and a battery that were modified using Adobe^®^ Photoshop^®^ Elements 8 to obtain correct and incorrect circuits (**Figure 1**). These images were designed to obtain various levels of certainty and uncertainty. Therefore, some of them were more intuitive and did not involve any known misconceptions, whereas others were less intuitive and involved common and well-known misconceptions. To ensure that these images involved misconceptions, a committee composed of two experienced teachers and two university professors in science education (conceptual change specialists, who have published in their field) participated in their creation and shortlisted the ones that were most likely to involve known misconceptions. For example, in **Figure 1**, circuit A is “correct” and can be considered as intuitive because it did not involve, according to the committee, any frequent misconceptions. Circuit B is “incorrect” and might involve the common misconception that only one wire is enough to light up a bulb (Pesman and Eryilmaz, 2010). Circuit C is “correct,” but many students usually get this question wrong, most likely because they believed the misconception that the electric current is “consumed” in the circuit (Çepni and Keleş, 2006) and therefore they believed that the first bulb (in the direction of current) should have been brighter than the second one. Circuit D is “incorrect” (because the lower extra wire produces a short circuit), but most participants get this question wrong, most likely because they believed the misconception that extra wires cannot affect circuits (Sencar and Eryilmaz, 2004).

Prior to the experiment, the bank of images of electric circuits was tested with 243 participants (with characteristics such as age, school level, and gender comparable to those of participants who were later tested in the MRI machine) in order to select a set of images that would optimize the chances of obtaining, with an equivalent set of participants, a sufficient number of certain and uncertain responses, and also to control some possibly confounding variables. Thus, the final selection of images for the cognitive task used in the fMRI optimized the possibility of obtaining balanced numbers of scientifically correct/incorrect circuits, as well as an approximately equivalent proportion of correct/incorrect answers and of certain/uncertain answers.

**FIGURE 2:**
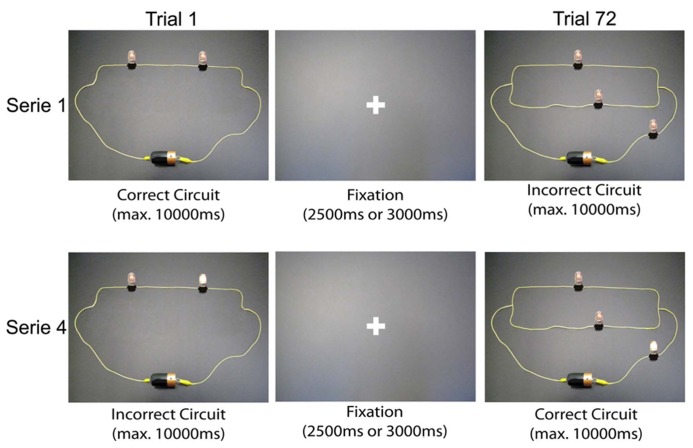
**Excerpts of the fMRI task, composed of 288 electric circuit images**.

The final selection of stimuli used during the fMRI sessions was composed of a set of 288 electric circuits divided into four equivalent series of 72 randomly presented trials (**Figure 2**). Each stimulus was presented for a maximum of 10000 ms (this presentation ended automatically when the participant pushed the button to answer), followed by a fixation period of 2500 ms (for half of the stimuli) or 3000 ms (for the remaining half). For each circuit, participants had to push one of the following four buttons: (1) “The circuit is correct; I am certain.” (right index finger); (2) “I think the circuit is correct, but I am uncertain.” (right middle finger); (3) “The circuit is incorrect; I am certain.” (left index finger); and (4) “I think the circuit is incorrect, but I am uncertain.” (left middle finger). Depending on the accuracy of the answers, these four possibilities allowed us to classify each answer in one of the four possible response categories (OE, UE, LC, and LD).

### PROCEDURE

After they gave their written informed consent, participants were taken to a simulation room with a computer on a desk and a MRI Simulator^TM^ (Psychology Software Tools, Inc.). Sitting at the desktop computer, they had to read the instructions for the task and do a practice task, which was composed of 20 electric circuits similar to those used in the fMRI task. Afterward, the participants repeated the practice task, but in the MRI simulator. Participants were, at that time, explicitly informed not to move during the practice task and imaging acquisition. Immediately afterward, the task was administered in the real fMRI machine. Structural images were also obtained at the end of the four functional image series.

### IMAGE ACQUISITION

Imaging was performed in a Siemens 3.0 Tesla MAGNETOM Trio TIM using a 32-channel head coil. Functional images were obtained with a gradient echo EPI sequence (TR = 2000 ms, TE = 30 ms, FA = 90°, matrix size = 64 × 64, voxel size = 3 mm × 3 mm × 3 mm, number of slices = 33, slice gap = 25%, interleaved, AC-PC line orientation, whole brain scanned). The first two images were automatically eliminated by the system. Structural images were obtained with a MPRAGE sequence (TR = 2300 ms, TI = 900 ms, TE = 2.98 ms, FA = 9°, matrix size = 256 × 256, voxel size = 1 mm × 1 mm × 1 mm, number of slices = 176, interleaved, sagittal orientation). Head motion was minimized by cushions arranged around each participant’s head. Stimuli were presented with E-Prime 2.0 software (Psychology Software Tools, Inc.) via a mirror and a projection system. Subjects’ responses were collected with the Fiber Optic Button Response System (Series 1) from Psychology Software Tools, Inc.

### STATISTICAL ANALYSIS

Data analysis was performed using SPM8 (Wellcome Department of Imaging Neuroscience, London, UK). Each participant’s functional data were motion-corrected (realignment with mean image), spatially normalized (into the standard MNI space using the segmentation method in SPM8), and smoothed (using a Gaussian kernel of 8 mm FWHM). The general linear model (GLM) was used for modeling the data. More precisely, trial-related activity was modeled by convolving a vector of trial onsets with a canonical hemodynamic response function (HRF). The six movement parameters were also included in the model as regressors of no interest.

Three of the 22 subjects had one functional series with an empty condition because they were always sure (or unsure) of their answers for all the electric circuits in a series. These three series were excluded from the analysis.

## RESULTS

### BEHAVIORAL RESULTS

Responses to a total of 5976 electric circuits were analyzed. Twenty of them (0.3%) remained unanswered by participants. Participants reported to be certain of 64.7% of their answers and uncertain of 35.0%. Behavioral task results (**Table 1**) show that the average reaction time was longer when participants reported being uncertain of their answer (*M* = 3911 ms; SD = 1730 ms) compared to when they were certain (*M* = 3091 ms; SD = 1730 ms). This difference (820 ms) is statistically significant [*t*(5954) = -18.06, *p* < 0.001].

**Table 1:** Overview of the behavioral task results.

	Uncertainty	Certainty
Mean reaction time	3911 ms	3091 ms
Standard deviation	1730 ms	1561 ms
Total number of stimuli	2091	3865
Number of stimuli by type of electric circuit
Scientifically correct circuit	1060	1750
Scientifically incorrect circuit	1031	2115
Number of stimuli by students’ answer
“The circuit is correct”	945	1881
“The circuit is incorrect”	1146	1984
Number of stimuli by value of the answer
Right answer	1030	2178
Wrong answer	1061	1687
Number of stimuli by gender
Men	891	1978
Women	1200	1887
Number of stimuli by series
Series 1 and 2	1084	1853
Series 3 and 4	1007	2012

*Mean reaction time is significantly longer when students are uncertain of their answers compared to when they are certain, *t*(5954) = -18.06, *p* < 0.001.*

The stimuli for which participants claimed to be uncertain of their answers were composed of a similar number of scientifically correct and incorrect electric circuits (50.7 and 49.3%, respectively), right and wrong answers (49.3 and 50.7%), and men’s and women’s answers (42.6 and 57.4%). There was also a comparable number of stimuli that were evaluated as being correct and incorrect by participants (45.2 and 54.8%), and there were as many uncertain stimuli in the first two series of the session as there were in the last two (51.8 and 48.2%).

The stimuli for which participants claimed to be certain of their answers were also composed of a similar number of correct and incorrect electric circuits (45.3 and 54.7%, respectively), right and wrong answers (56.4 and 43.6%), and men’s and women’s answers (52.2 and 48.8%). Furthermore, there was a comparable number of stimuli that were evaluated as being correct and incorrect by participants (48.7 and 51.3%), and there were as many uncertain stimuli in series 1 and 2 as there were in series 3 and 4 (47.9 and 52.1%).

These results enable us to assume that the obtained results cannot be attributed to unbalanced quantities of particular types of answers, as would be the case if we had a prevalence of the following types of answers: masculine (vs. feminine), associated with scientifically correct circuits (vs. incorrect), answered as correct (vs. answered as incorrect), right (vs. wrong), or chronologically presented at the beginning of the task (vs. at the end of the task).

### fMRI RESULTS

#### Research question No.1 (uncertainty vs. certainty)

**FIGURE 3:**
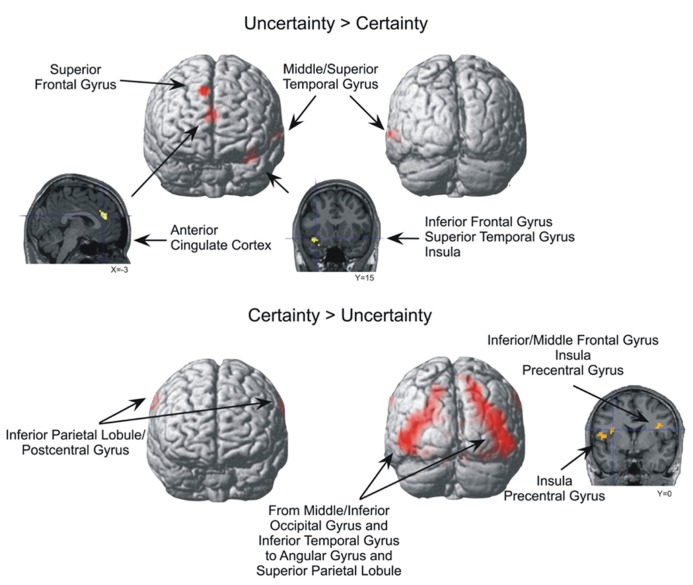
**Brain areas significantly more activated in the *Uncertainty > Certainty* and *Certainty > Uncertainty* contrasts (*p* < 0.0005, uncorrected, min. 20 voxels**).

For the Uncertainty > Certainty contrast, four brain areas were significantly more activated (*p* < 0.0005, uncorrected, min. 20 voxels) when participants expressed uncertainty about their answers compared to when they expressed certainty (**Table 2** and **Figure 3**). The most posterior activation was located at the intersection of the left middle/superior temporal gyrus (BA 21/22), while the most anterior activation was located in the right superior frontal gyrus, extending to the dorsomedial frontal cortex (BA 8/9). Two other significant activations were recorded. One was located at the intersection of the left anterir insula, superior temporal gyrus, and inferior frontal gyrus (BA 13/38/47), while the other was located in the right and left anterior cingulate cortex (ACC; BA 24/32). No significant activation was noted in the parietal and occipital lobes.

**Table 2:** Overview of the neuroimaging results (*p* < 0.0005, uncorrected, min. 20 voxels, MNI coordinates in mm, L= left, R = right, LR = left and right).

Regions	*k*	*x*	*y*	*z*	*t*
**Uncertainty > Certainty**
L middle/superior temporal gyrus (BA 21/22)	41	-63	-39	3	5.36
R superior frontal gyrus (BA 8/9)	34	9	54	45	5.32
LR anterior cingular cortex (BA 24/32)	59	-3	36	21	4.86
L inferior frontal gyrus/superior temporal gyrus and insula (BA 47/38/13) – into the lateral sulcus	27	-36	15	-12	4.49
**Certainty > Uncertainty**
LR from middle/inferior occipital gyrus and inferior temporal gyrus (BA 19/18/37, L peak) to angular gyrus (BA 39) and superior parietal lobule (BA 7/19)	1545	-45	-72	-3	8.40
LR inferior parietal lobule and postcentral gyrus (BA 40/2)	83	63	-24	42	6.31
R inferior/middle frontal gyrus (BA 44/8), insula (BA 13), and precentral gyrus (BA 4/6)	40	42	-3	24	5.27
L insula (BA 13) and precentral gyrus (BA 4/6)	38	-33	0	18	5.19
L insula (BA 13) and precentral gyrus (BA 4/6)	22	-48	0	6	4.62

For the Certainty > Uncertainty contrast, a large bilateral activation, beginning in the middle/inferior occipital gyrus and the inferior temporal gyrus and ending in the angular gyrus and superior parietal lobule, was observed when the participants were certain of their answers compared to when they were uncertain (**Table 2** and **Figure 3**). The activation in the left middle/inferior occipital gyrus and inferior temporal gyrus survived to a threshold of *p* < 0.05, FWE-corrected, min. 20 voxels. Other significant activations were observed bilaterally at the intersection of the inferior parietal lobule and the postcentral gyrus, and at the intersection of the precentral gyrus and the insula. A part of the activation at the intersection of the right precentral gyrus and the insula also reached the right inferior/middle frontal gyrus. The L middle/inferior occipital gyrus/inferior temporal gyrus (BA 19/18/37) activation in the Certainty > Uncertainty contrast survived to a threshold of *p* < 0.05, corrected, min. 20 voxels (with MNI coordinated of -45 -72 -3).

#### Research question No.2 (legitimate certainty vs. over-estimation)

**FIGURE 4:**
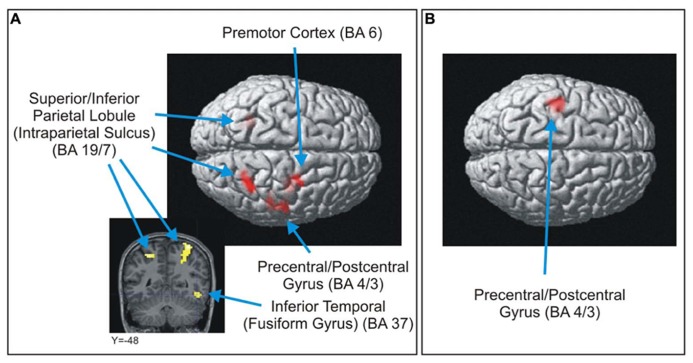
**Brain areas significantly more activated in the *LC > OE* (A) and *OE > LC***(B)** contrasts (*p* < 0.0005, uncorrected, min. 20 voxels**).

For the LC > OE contrast, five brain areas were significantly more activated (*p* < 0.0005, uncorrected, min. 20 voxels) when participants answered correctly and were certain of their answers compared to when they were equally certain, but answered incorrectly [**Table 3** and **Figure 4** (images of the left part of the figure)]. These regions were the left and right intraparietal sulcus, the right premotor cortex, the right fusiform gyrus and the right motor cortex. This last activation was also recorded (this time on the left part of the brain) in the OE > LC contrast [**Figure 4** (image of the right part of the figure)].

**Table 3:** Overview of the neuroimaging results (*p* < 0.0005, uncorrected, min. 20 voxels, MNI coordinates in mm, L = left, R = right, LR = left and right).

Regions	*k*	*x*	*y*	*z*	*t*
**Legitimate certainty > Over-estimation**
L superior/inferior parietal lobule (L intraparietal sulcus; BA 19/7)	26	-27	-48	48	5.64
R premotor cortex (BA 6)	87	21	-9	45	5.51
R superior/inferior parietal lobule (R intraparietal sulcus; BA 19/7)	102	33	-51	63	5.50
R inferior temporal gyrus (fusiform gyrus; BA 37)	34	48	-48	-9	4.91
R precentral/postcentral gyrus (motor cortex; BA 4/3)	43	51	-21	51	4.65
**Over-estimation > Legitimate certainty**
L precentral/postcentral gyrus (motor cortex; BA 4/3)	72	-42	-18	57	5.78

#### Other results

All contrasts related to Certainty > Uncertainty (LC > UE; LC > LD; OE > UE; OE > LD) showed patterns of activation that we also found in the general Certainty > Uncertainty contrast (i.e., occipito-parietal activations). None of the other possible contrasts between the four response categories (UE > LD; LD > UE; LD > OE; UE > LC) recorded significant activation, except for UE > LC. This particular contrast recorded a significant activation of the anterior cingulate cortex. (MNI coordinates: *k* = 51; *x* = -3, *y* = 30, *z* = 30; *t* =4,93; *p* < 0.0005, uncorrected, min. 20 voxels)

## DISCUSSION

### CERTAINTY AND UNCERTAINTY IN A MULTIPLE-CHOICE TASK INVOLVING MISCONCEPTIONS

#### Uncertainty

Our hypothesis for Uncertainty > Certainty included the activation of the anterior cingulate, the superior frontal gyrus and the posterior fronto-median cortex (BA8; with particular attention to medial temporal activations). It appears that much of this hypothesis is confirmed, since these regions were activated, with few unexpected activations (see below). We therefore believe that our results are in line with results obtained in other studies that were looking for uncertainty caused by lacks of knowledge.

Indeed, the ACC which has been reported in a number of related studies (Critchley et al., 2001; Krain et al., 2006; Harris et al., 2008; Daniel et al., 2010; Stern et al., 2010) was activated. There are at least two broad perspectives on the role of this region in decision making. The first focuses on the fact that the ACC is often involved in tasks in which detection of conflicts (often called *error detection* or *conflict monitoring*) is necessary. These tasks include the Stroop task (e.g., Bench et al., 1993; Bush et al., 1998), various versions of the Flanker task (e.g., Botvinick et al., 1999), tasks related to the go/no-go paradigm (e.g., Braver et al., 2001), and tasks where prepotent responses have to be overcome (e.g., Nathaniel-James et al., 1997). The second perspective associates the ACC with the evaluation of the action outcomes. It was founded on a group of studies showing that the ACC is engaged when negative outcomes are expected, such as monetary loss in gambling tasks (Bush et al., 2002), negative feedback (e.g., van Duijvenvoorde et al., 2008), or social rejection (Eisenberger et al., 2003). According to this perspective, it has been argued that the ACC guides decision making by performing a cost-benefit analysis that may be based on past action outcomes. These two perspectives are not necessarily in competition with each other. Indeed, Botvinick (2007) proposed an integrative account of the role of the ACC. He argued that detection of conflicts can contribute to decision making (1) by sending signals announcing the necessity of better cognitive control in the prefrontal cortex, but also (2) by provoking a kind of negative reinforcement leading one “to avoid tasks or strategies that have given rise to it in the past” (p. 359). This second contribution explains how the detection of conflict can be linked to negative outcomes. However, in our task, there were no negative outcomes or feedback. Therefore, since participants reported being uncertain about some of their answers, it is possible that there experienced conflicts. These might have been caused by an incompatibility between knowledge about electric circuits learned in school and misconceptions that continued to drive decision making. Another hypothesis would be that conflicts might have been caused by competition between more than one conception, or conflicts between different conceptions applying to different parts of the circuits.

Also, in compliance with our hypothesis, the superior frontal gyrus and dorsomedial prefrontal cortex were activated. The superior frontal gyrus has often been reported in other studies about decision making under uncertainty (Henson et al., 2000; Volz et al., 2004, 2005; Tobler et al., 2007; Harris et al., 2008; Daniel et al., 2010; Hosseini et al., 2010; Stern et al., 2010). This region, more specifically the dorsolateral prefrontal cortex, is known to be involved in working memory and response selection (Rowe et al*.,* 2000; Miller and Cohen, 2001; Curtis and D’Esposito, 2003; Blumenfeld and Ranganath, 2006). When our subjects were uncertain of their answers, they might have been hesitating between two competitive responses (“Is the circuit correct or incorrect?”) and possibly between two levels of certainty (“Am I sure or not of my answer?”). Mean reaction times might have been longer for uncertain than for certain responses (3.911 ms compared to 3.091 ms) as a result of such hesitations. In a more pedagogical interpretation, we suggest that participants might have been hesitating between different conceptions that coexist and compete and that it took more time to answer because of the need to suppress some interfering conceptions (Shtulman and Valcarel, 2012). The dorsomedial prefrontal cortex has also been associated with uncertainty in decision making in a number of studies (Grinband et al., 2006; Platt and Huettel, 2008; Zaretsky et al., 2009; Daniel et al., 2010; Hosseini et al., 2010; Stern et al., 2010). The role of this region in decision making is not completely understood, but is perhaps related to uncertainty independent of the kind of uncertainty involved in the task. In a series of experiments, Volz et al. (2003, 2004, 2005) tried to find differences in brain activation between uncertainty caused by a lack of knowledge and uncertainty caused by the manipulation of the probability of events. In both situations, the dorsomedial prefrontal cortex was recruited.

Another region that is more activated under uncertainty is the left superior temporal gyrus, extending to the middle temporal gyrus. Contrary to the brain areas previously discussed (ACC, insula, and superior/dorsomedial frontal cortex), the activation of this region has been rarely reported in studies about decision making under uncertainty, and when it has been, the results were contradictory. For example, Stern et al. (2010) observed that the left and right middle temporal gyrus regions are activated during the execution of a decision made under uncertainty, and Simmons et al. (2008) observed that the intolerance of uncertainty is positively correlated with the activation of the right superior temporal gyrus. However, other studies have presented opposite results. For example, Schlosser et al. (2009) noted that the right middle temporal gyrus is negatively correlated to uncertainty, and Huettel et al. (2005) observed that the activation of the left superior temporal gyrus and the right middle temporal gyrus is independent of the level of uncertainty. These contradictory studies point to the idea that the left middle and superior temporal gyrus activations observed in our data are perhaps not directly related to uncertainty. Indeed, since the left temporal lobe is known to be involved in language processing and semantic and declarative memory, it is possible that the uncertainty trials require the activation of an inner language. Such an inner language might need to be used by learners during the resolution of complex problems that involve many elements linked in complex ways, like in our electric circuits, especially the most complex ones. It is also possible that the use of this inner language might be an intermediate state of knowledge, before automatization is achieved, and therefore should be encouraged in the context of learning how to resolve complex problems in class.

We also recorded activation of the insula (extending to the inferior/middle frontal gyrus and the precentral gyrus). This area has also been activated in a number of studies about decision making under uncertainty (Volz et al., 2003, 2005; Huettel et al., 2005; Grinband et al., 2006; Platt and Huettel, 2008; Preuschoff et al., 2008; Simmons et al., 2008; Daniel et al., 2010; Hosseini et al., 2010; Sarinopoulos et al., 2010; for a review, see Singer et al., 2009), and is associated with aversive (Sarinopoulos et al., 2010) emotional response (Bechara and Damasio, 2005), sometimes in risky situations (Platt and Huettel, 2008; Preuschoff et al., 2008). But the insula is also, in some cases, associated with cognitive functions, like differentiation (Kurth et al., 2010), depending on its distinct sub-regions. Since its activation was not necessarily excepted and because of its numerous possible functions that were difficult to relate to our task, we will propose a rather conservative interpretation: since, in our study, the subjects did not receive any negative feedback, nor were they subjected to aversive stimuli or risky gain/loss situations, we suggest that being uncertain, even in the absence of incentives or feedback (Stern et al., 2010), may cause an intrinsic negative emotional arousal. These results tend to support the idea that uncertainty, disequilibrium or cognitive dissonance (Festinger, 1957) are uncomfortable states that could explain avoidance, and, in the long run, loss of interest. However, since the insula was also activated in the Certainty > Uncertainty contrast, it is likely that it is essentially the properties of our task that involved this region, possibly because of its complexity (integration/differentiation of many elements of the electric circuits).

#### Certainty

We did not formulate explicit hypotheses about the Certainty > Uncertainty contrast, since the studies we cited in the previous section did not extensively discuss the brain-based mechanisms related to certainty. There are still, however, a few studies that focus on the neural correlates of certainty associated with decision. For example, some researchers have studied the brain-based mechanisms of confidence in recognition memory (e.g., Chua et al., 2006, 2009; Kim and Cabeza, 2009; Hayes et al., 2011). Their results reveal that the MTLs, especially the hippocampal region which is known to be involved in memory, are more activated for high confidence. In our study, we did not observe any activation of this region probably because the electric circuit is not a recognition memory task, or because the activation happened equally in both response categories. We however observed significant activations in the lateral intraparietal cortex. More precisely, we found bilateral extended activations in the posterior region of the brain, beginning at the inferior occipital gyrus and ending at the angular gyrus and the superior parietal lobule. These regions are typically related to visuospatial processing. The ventral activations are likely related to visual processing and the identification of many of the objects in the stimuli (e.g., identification of the battery, bulbs, and wires) and complex configurations, and the more dorsal activations are probably related to spatial processing. This predominance of the visuospatial processing during trials where participants reported to be sure of their responses is probably caused by the fact that uncertain trials caused a larger amount of cognitive processing and, indeed, the subjects needed more time to answer uncertain trials than certain trials. Another interpretation, however, would be that since the “certainty” response category can likely be associated with cases where participants founded their answers on a strong enough conceptual basis (regardless of whether this basis was scientific or not), it is possible to suggest that conceptions about electric circuits could be grounded in visuospatial functions. Indeed, Kiefer and Pulvermüller (2012) have recently suggested, based on an extensive review of literature, that conceptual features “are stored in distinct sensory and motor brain areas depending on specific sensory and motor experiences during concept acquisition” (p. 805). For example, they found in studies that hand-related concepts like “pick” and mouth-related concepts like “lick” activate corresponding lateral and ventral parts of the motor cortex, whereas foot-related concepts like “kick” activate more dorsal parts (p. 812). According to such “embodiment theory” interpretations, the electricity concepts, at least in the context of our task, could be the consequence of our participants’ past experiences with real electric circuit tasks that required visuospatial treatment. These experiences might have been numerous enough to ultimately produce feelings of certainty. We believe that the activation of the most dorsal parts of the large posterior activation (involved in spatial processing) supports this interpretation, especially keeping in mind that in the “uncertain” response category, which is the contrasting condition, subjects also had to treat visual images of circuits.

It is true that our task presented images that were similar to realistic photographs of electric circuits and did not refer to abstract representations of circuits, such as mere schemas with straight lines, nor did it not use sentences to describe scientific phenomena or conceptions. Indeed, we believe that if our stimuli had been induced with words instead of images, by stating for example that “one wire is sufficient to light a bulb,” it would have been highly likely that visuospatial regions might not have been recruited as much. It does not appear unreasonable, however, to suggest that scientific competency (at least with electric circuits) might be associated with visuospatial treatment. Therefore, dealing with real-life tasks (like school lab experiments) and complex problems that necessitate visual treatment could improve the ease with which students learn about electrical concepts.

### NOVICES OVERCOMING – OR FALLING INTO – THE TRAP OF MISCONCEPTIONS

In accordance with previous research on the differences between novices and experts in scientific tasks involving misconceptions (see above Section “Difference Between Over-Estimation and Legitimate Certainty”), our second hypothesis suggested that contrasts between LC and OE (LC > OE) would show activations of the ACC and the dorsolateral and ventrolateral cortices. This hypothesis could not be confirmed. Typical inhibition mechanisms did not show any significant activation for *correct-and-certain* answers compared to *incorrect-and-certain* ones. Thus we cannot say that the brain activations of novices when they give correct and certain answers are similar or close to the brain activations of experts when they give correct answers. Therefore, it is possible that the development of a minimal level of expertise (unlike our participants, even though they sometimes produced correct answers) is required to record inhibitive activations. We believe that further investigation into this matter is required. Some recorded activations might be associated with the use of left and right hands (precentral/postcentral gyrus (motor cortex) [left for OE > LC and right for LC > OE]). Indeed, a *post hoc* analysis showed that in the LC response category, answers were expressed more often with the left hand (1,001 stimuli vs. 890), as OE showed a majority of right-hand answers (858 vs. 641). The right premotor cortex was also activated for LC > OE, most likely indicating that answering with the left hand required more control (Halsband et al., 1993), possibly because our participants were right-handed.

The activation of the left and right intraparietal sulcus that was recorded is usually associated with number processing (Dehaene et al., 2003; Houdé et al., 2011). We also recorded an activation of the fusiform gyrus that is usually associated with the identification of objects (Puce et al., 1996; Tarkiainen et al., 2002). In our task, these regions were activated when participants were able to avoid falling into the trap of misconceptions. It is therefore possible that better processing of the complexity of electric circuits (positioning and number of bulbs, wire, etc.) played an important role in understanding – and performing with – simple electricity problems. Therefore, merely directing more visual attention to these problems could explain the difference between success and failure.

## CONCLUSION

From an educational perspective, we first believe that presented results are rather encouraging because they suggest that resolutions of multiple-choice educational tasks could be understood as decision-making processes. Second, since during our task we never gave any negative feedback to the participants, we believe that activations that occur during uncertainty can be associated with an internal conceptual conflict between competing conceptions. It is also possible that the activation of the left temporal lobe, and the corresponding suggestion that inner language was used, could be an indication of a conflict that would require a heavier dialectic process to be resolved. We believe that these are interesting results because similar interpretations have been proposed, but for differences between experts and novices (Dunbar et al., 2007; Masson et al., in press). It is therefore possible to believe that these expert > novice contrasts have revealed activations that are in fact attributable to typical uncertain > certain contrasts. However, this would either suggest that novices’ uncertainty implies the availability of more conceptions (than in “certain” situations) to confront, or that experts are typically more inclined to uncertainty than novices. A possible educational interpretation of the presence of conflict in uncertain situations might also bring support to pedagogical models that believe that many conceptions about a single phenomenon can coexist and be conflicted, instead of models that believe that only one conception can exist at a time for a given phenomena. Many conceptual change models, for instance, postulate that learning consists of modifying or restructuring initial conceptions. Instead, it could be hypothesized that conceptual change is often a modification of the relative statuses of some conceptions at the expenses of others, until one of them prevails. We have also suggested that because of the activation of the anterior insula, uncertainty appears to be a rather uncomfortable state, and even if cognitive conflict is generally considered useful for learning (accommodation), it remains unsettling.

For the “certainty” category of responses, we found large posterior activations, usually associated with visual and spatial processing. Our interpretation suggests that scientific conceptions about electricity might be grounded in the visuospatial circuits of the brain. Even if other means should not be discarded as interesting ways to develop scientific conceptions, we hypothesize, that visuospatial training with real (or realistic) tasks (such as laboratory tasks or working with realistic images), might lead to more confidence when tackling tasks like the one we used in our study. We believe that this suggestion is quite in line with Uttal et al.’s (2012) results which were obtained with an extensive meta-analysis: “Spatial training programs therefore may play a particularly important role in the education and enhancement of spatial skills and mathematics and science more generally” (p. 19). This result might be the most robust we have presented in this article, because the recorded large posterior activation within the Certain > Uncertain contrast is the only one that survived a corrected threshold.

Our second hypothesis was that we would find typical inhibition activations in the contrast between correct-and-certain answers and incorrect-and-certain ones (LC > OE). In this setup, we hypothesized that correct-and-certain answers would be typical of expert answers. However, we were not able to show typical expert or inhibitive activations. Instead, most recorded activations could be interpreted as the use of left and right fingers, and possibly the mobilization of identification and number processing. Based on these results, we can hypothesize that correct answers might require a more thorough examination of our electrical circuits. Indeed, some were rather complex and might have necessitated a greater number of verifications (identification and enumeration of all different parts) and therefore the mobilization of visual attention resources. It can also be suggested that in order to answer correctly to scientific questions, novices recruited resources that differed from the ones experts would recruit.

This research can be considered as an effort to link neuroscientific and educational knowledge through the use of an authentic educational context involving the resolution of a multiple-choice task (considered as a decision-making process). We believe that this effort has been fruitful because the recorded activations about uncertainty/certainty were typical of decision-making processes that involved uncertainty caused by “lacks of knowledge.” Therefore we believe that many of the extended knowledge elements about decision making might prove useful in the long run to better understand school performance and failure. But we also believe that much more work has to be done in order to better understand the differences between the production of correct answers and expertise, and also the origins and the function of uncertainty in learning. In science, uncertainty can indeed be the driving force of knowledge development, but in some cases, it can also be paralyzing. Thus expert uncertainty might be at least somewhat different from novice uncertainty.

## Conflict of Interest Statement

The authors declare that the research was conducted in the absence of any commercial or financial relationships that could be construed as a potential conflict of interest.
